# Distributional consequences of including survivor costs in economic evaluations

**DOI:** 10.1002/hec.4401

**Published:** 2021-07-30

**Authors:** Klas Kellerborg, Werner Brouwer, Matthijs Versteegh, Bram Wouterse, Pieter van Baal

**Affiliations:** ^1^ Erasmus School of Health Policy & Management Erasmus University Rotterdam Rotterdam Netherlands; ^2^ Institute of Medical Technology Assessment Erasmus University Rotterdam Rotterdam Netherlands

**Keywords:** economic evaluation, future costs, inequalities in health

## Abstract

Medical interventions that increase life expectancy of patients result in additional consumption of non‐medical goods and services in ‘added life years’. This paper focuses on the distributional consequences across socio‐economic groups of including these costs in cost effectiveness analysis. In that context, it also highlights the role of remaining quality of life and household economies of scale. Data from a Dutch household spending survey was used to estimate non‐medical consumption and household size by age and educational attainment. Estimates of non‐medical consumption and household size were combined with life tables to estimate what the impact of including non‐medical survivor costs would be on the incremental cost effectiveness ratio (ICER) of preventing a death at a certain age. Results show that including non‐medical survivor costs increases estimated ICERs most strongly when interventions are targeted at the higher educated. Adjusting for household size (lower educated people less often live additional life years in multi‐person households) and quality of life (lower educated people on average spend added life years in poorer health) mitigates this difference. Ignoring costs of non‐medical consumption in economic evaluations implicitly favors interventions targeted at the higher educated and thus potentially amplifies socio‐economic inequalities in health.

## 1 INTRODUCTION

When medical interventions postpone death, costs arise in added life‐years due to consumption of medical and non‐medical goods (Meltzer, 1997). Whether or not to include these costs in economic evaluations conducted from a societal perspective remains an issue of controversy (de Vries, van Baal, & Brouwer, 2018). Many national guidelines for economic evaluation currently do not recommend the inclusion of survivor costs (ISPOR, 2018). However, the recently updated, influential US guidelines do specifically recommend their inclusion (Sanders et al., 2016). The few studies that investigated the impact of inclusion of future non‐medical costs on the ICERs of lifesaving interventions show it can be substantial (Kruse, Sørensen, & Gyrd‐Hansen, 2012; Manns, Meltzer, Taub, & Donaldson, 2003; Meltzer, 1997; Meltzer, Egleston, Stoffel, & Dasbach, 2000).

Many countries adopting a societal perspective include production gains in economic evaluations but exclude costs of non‐medical consumption (ISPOR, 2018). This difference can be considered inconsistent, since many of the theoretical arguments (not) to include non‐medical consumption also pertain to production (Meltzer, 1997; Nyman, 2004). Moreover, this practice of including production gains but excluding non‐medical consumption has potential distributional consequences: it benefits the higher socio‐economic groups, who are the most productive (Meltzer, 1997) but also have the highest non‐medical consumption across the lifecycle (Attanasio & Pistaferri, 2016; Ferná ndez‐Villaverde & Krueger, 2007).

This paper estimates the distributional consequences of including non‐medical consumption costs in economic evaluations across groups with different socio‐economic status (SES). Doing so, we add an important element to the literature on cost‐effectiveness and inequality. There is a growing interest in distributional consequences in cost‐effectiveness evaluations, and many health policies are explicitly targeted at reducing inequalities in health (Cookson et al., 2017; Asaria et al., 2016). As health problems vary strongly with socio‐economic status (Cutler & Lleras‐Muney, 2010; Meara, Richards, & Cutler, 2008; Smith, 1999), the relevant future non‐medical costs incurred are likely to differ per intervention or even target group. Using a rich dataset from The Netherlands, we estimate age profiles of consumption by education.

In addition, we adjust for differences in household size across educational groups at different ages. The (marginal) costs of non‐medical consumption are lower for individuals in multi‐person households than for singles, as the first group benefits from economies of scale (Kellerborg et al., (submitted)). Due to lower life expectancy of lower educated, they are at greater risk of living alone at older age relative to their higher educated counterparts (Hagenaars et al., 1994). We also account for the fact that the quality of life of persons from low SES groups in general is lower than that of people from high SES groups (Gheorghe et al., 2016). This is important, as the impact of including future costs is stronger when life years are gained in poor health.

## 2 METHODS

### 2.1 Data

**FIGURE 1:**
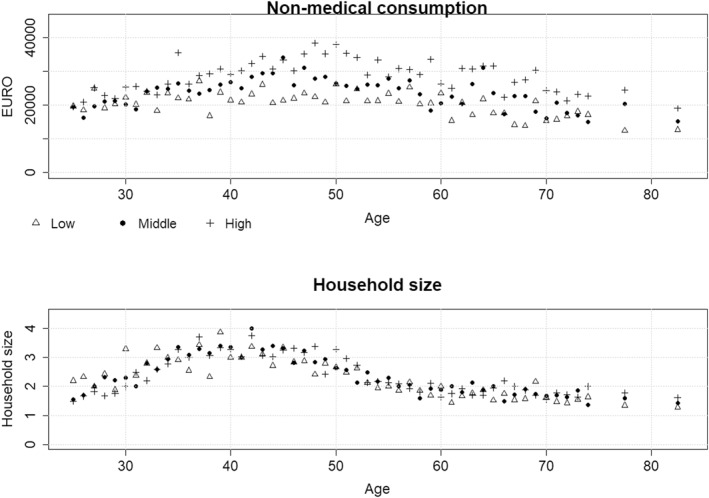
Non‐medical consumption per household (top panel) and household size (bottom panel) by education and age. Non‐medical consumption is shown in 2017 EURO prices

We used data from the Dutch budget survey (Budgetonderzoek) from the years 2003 and 2004 which is a yearly cross‐sectional survey collected among the non‐institutionalized population of the Netherlands. The households taking part in the survey report on a comprehensive set of consumption categories (e.g. consumption related to eating, transportation, housing, vacation) using diaries which were collected by interviewers on a regular basis. We removed all medical consumption of the household and adjusted the data to 2017 prices. Age was defined as the age of the household head. Educational attainment was determined by the highest educational attainment of the household head and categorized in three categories: low, middle and high (for more details on the data and methods see the online Supplementary File). Figure 1 gives an overview of the data.

### 2.2 Model specification

Our approach to estimating the impact of education on the ICER consists of several steps. First, we translate household consumption into per household equivalent consumption using the OECD‐modified equivalence scale (Hagenaars et al., 1994). This scale assigns a weighting factor per additional individual in a household of 0.5 for each adult and 0.3 per child. We then estimated log scaled household equivalent consumption as a function of age, stratified by education with the following model:

(1) ln(hhequiv.)=S(age⋅edu)+ε.

where *Hh equiv*. denotes annual non‐medical consumption per household equivalent. We used cubic P‐Splines to model the non‐linear age pattern with an interaction term for education and ε is a normally distributed error term (Eilers & Marx, 1996).

Given that we want to estimate the average costs of non‐medical consumption in case of a prevented death, we need to know the average household size at different ages for different educational classes. Preventing a death in a single person household will result in more additional consumption than preventing one in a multi‐person household. Therefore, we also estimated the probability of a household having more than one adult as a smooth function of age and education using a binomial logistic P‐splines model:

(2) $$p\left(Y\;=\;adults\;>\;1\right)=\frac{\text{exp}\left(S\left(age\cdot edu\right)+\varepsilon \right)\;}{1+\text{exp}\left(S\left(age\cdot edu\right)+\varepsilon \right)\;}$$

As in the first model, we apply P‐Splines on age with education as an interaction term. The average costs of non‐medical consumption caused by living one year longer by age and education (*nmc(age,edu)*) are then calculated as in Equation (3):

(3) $$\begin{array}{ccc} nmc\left(age,edu\right) & = & p\left(adults\;in\;hhs>\left.1\right|age,edu\right)\;\times \;hh\;equiv\left(age,edu\right)\;\times \;0.5\\ & & +\;\left(1-p\left[adults\;in\;hhs>\left.1\right|age,edu\right]\right)\;\times \;hh\;equiv\left(age,edu\right) \end{array}$$

The first part denotes the consumption for individuals in households with more than one adult times the probability of a death being prevented in a multi‐person household. The second part represents the consumption for individuals in single households times the probability of a death being prevented in a single‐person household.

### 2.3 ICER calculations

Equation (4) shows the elements included in the ICER when both production gains and costs of non‐medical consumption costs are taken into account:

(4) $$ICER=\frac{\Delta medical\;costs+\Delta production}{\Delta QALY}+\frac{\Delta nmc}{\Delta QALY}.$$

To calculate the impact on the ICER we focus solely on the second part of this equation: the additional costs from non‐medical consumption over the quality‐adjusted life year (QALY) gained $\left(\frac{\Delta nmc}{\Delta QALY}\right)$ from saving a life at different ages. These estimations may then be added to the ICER of a life prolonging intervention. $\frac{\Delta nmc}{\Delta QALY}$ was calculated in the following manner:

(5) $$\frac{\Delta nmc}{\Delta QALY}\;=\;\frac{\sum_{a}\left\{L\left(age=a,edu\right)\;\times \;nmc\left(age=a,edu\right)\right\}}{\sum_{a}\left\{L\left(age=a,edu\right)\times QoL\left(age=a,edu\right)\right\}}$$

where L(age=a,edu) is the number of years lived at age a for a particular educational group and Q(age=a,edu) is the average quality of life at age a for a particular educational group. Estimates of L(age,edu), QoL(age,edu) were taken from a study that estimated the quality of life and mortality in the Netherlands stratified by education (Gheorghe et al., 2016). We estimated the additional costs separately for the ages 25 to 82.5 over a lifetime horizon. We present estimates for deaths prevented in an average household but also for deaths prevented in a single‐person household. When calculating the costs for a death prevented in a single person household, we use one full household equivalent by age and education times added survival. Costs were discounted at 4 percent and effects at 1.5 percent in accordance with the Dutch guidelines (Zorginstituut Nederland, 2016). The ICERs as calculated using equation (5) can be interpreted as the cost effectiveness of hypothetical interventions in which a death at a certain age is prevented at zero intervention costs. Previous research (e.g. Meltzer, 1997; Kellerborg et al., 2020) has shown that such ICERs give a good indication of what the impact is of including future costs on the ICER of non‐hypothetical interventions.

Using Equations (3), (4) and 5, the influence of the three mechanisms that affect the ICER for different SES groups can be illustrated. First, equation (4) shows that including non‐medical consumption increases the numerator of the ICER, resulting in an increase of the ICER. As non‐medical consumption is lower for low SES groups than for high SES groups, including these survivor costs is *relatively* favorable for the interventions targeted at the low SES groups (although it increases the ICER for all interventions). Second, the higher educated enjoy both lower mortality rates as well as a higher quality of life resulting in a higher quality‐adjusted life expectancy at all ages (Gheorghe et al., 2016). Equation (5) highlights that lower quality of life values for the lower educated imply that non‐medical consumption is divided by a smaller number and hence a relative increase of the ICER. Third, accounting for differences in household size across SES groups involves accounting for the fact that low SES groups are less often part of multi‐person households. Thus, they benefit less often from economies of scale than high SES groups (Equation 3). This leads to relatively higher ICERs for interventions aimed at low SES groups.

## 3 RESULTS

**FIGURE 2:**
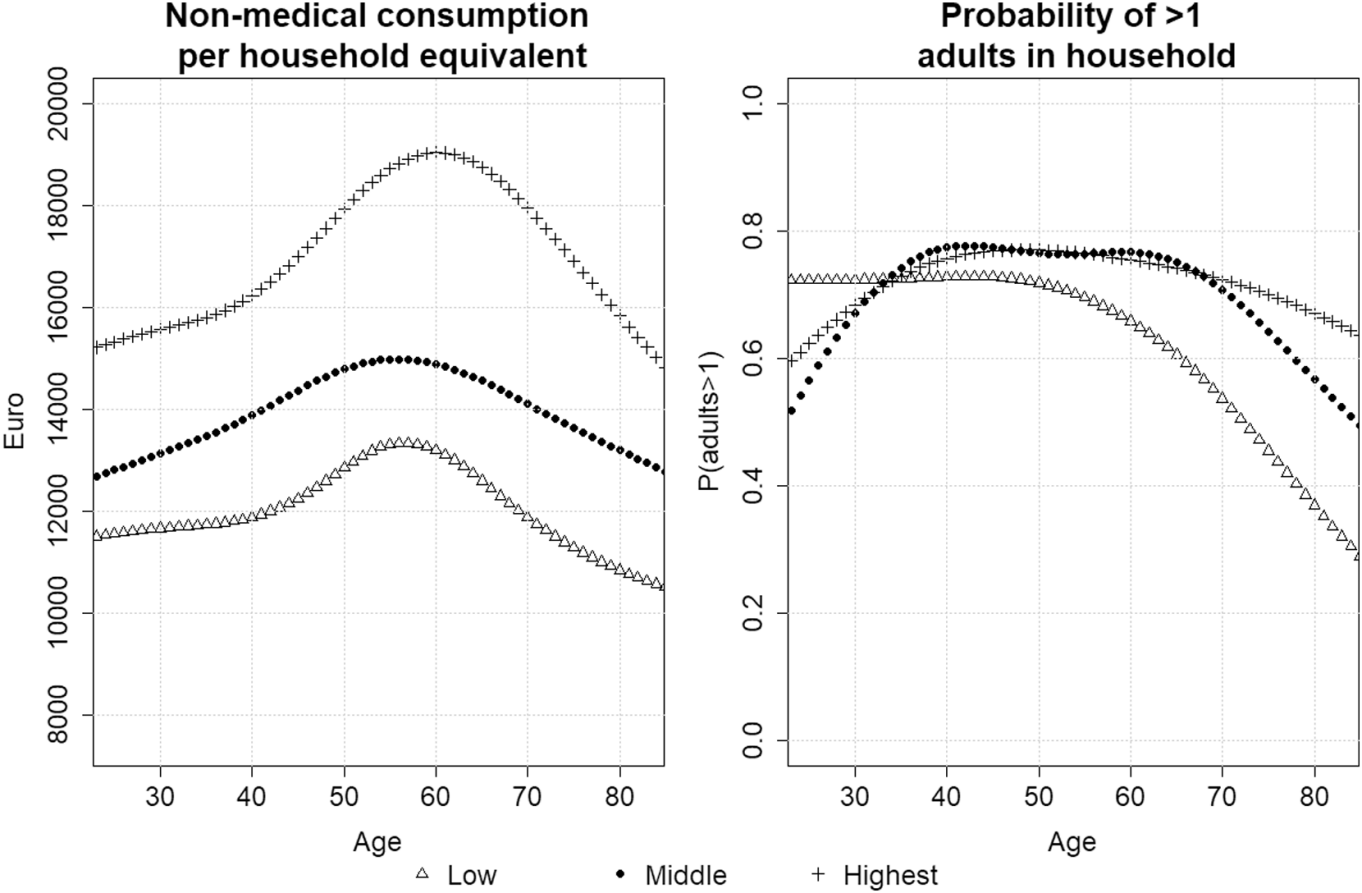
Prediction of non‐medical consumption by household equivalent (left) by education and age. Prediction of probability of households having more than one adult by age and education (right)

Figure 2 shows the predictions of the regression models to illustrate the impact of education and age on consumption and household size.

**FIGURE 3:**
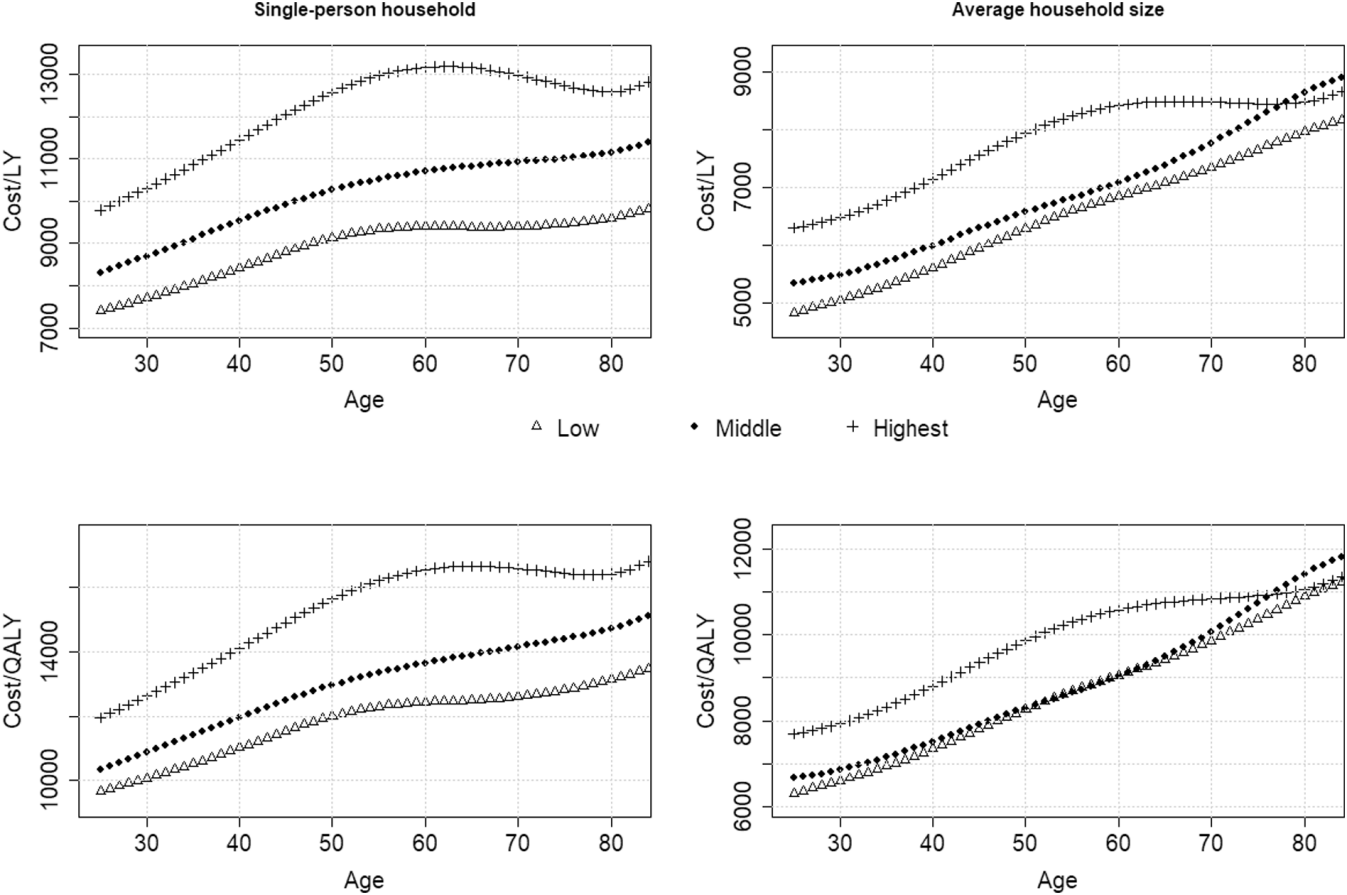
Impact on the ICER when saving a life at various ages by education. Left panels show predictions for preventing a death in a single‐person household and the right panels show predictions for preventing a death in an average household, top panels show estimations calculated with LYs and bottom panels with QALYs. Costs were discounted at a 4% annual rate and health effects at a 1.5% annual rate

Figure 3 displays the impact of including non‐medical survivor costs on the ICER by educational attainment. To better understand the effect of the different mechanisms, the ICER is calculated for preventing a death in a single‐person household as well as preventing a death in a household of average size (by age and education as estimated in Equation (3)), and using Life Years (LY) or QALYs in the denominator. The impact of survivor costs on the ICER differs substantially between the educational groups when we do not account for differences in household size. Using QALYs instead of life‐years as outcome increases the impact on the ICER, but it does not substantially affect the (absolute) differences across education groups. Controlling for household size decreases the impact on the ICERs, as well as the differences between the education groups. This can be seen by comparing the left panels in Figure 3 for single‐person households with the right panels in which we made predictions for an average household size using Equation (3). Especially at high ages, the high educated on average live in larger households (as shown in Figure 2) which results in a stronger decrease of the impact on the ICER than for the low educated.

To further illustrate the differences across model specifications we summarized the impact on the ICER for selected ages and assumptions in Table 1.

**TABLE 1:** Impact of including future non‐medical costs on the ICER

Household	Age	Educational Attainment	ΔCosts	ΔLY	ΔQALY	ΔCost/ΔLY	ΔCost/ΔQALY
Average household	30	Low	169,000	33.45	25.59	5100	6600
		Middle	193,300	35.28	28.25	5500	6800
		High	237,200	36.51	29.81	6500	8000
	65	Low	104,400	14.76	11.08	7100	9400
		Middle	117,500	16.23	12.64	7200	9300
		High	147,500	17.34	13.69	8500	10,800
	85	Low	39,700	4.84	3.52	8200	11,300
		Middle	45,600	5.44	4.09	8400	11,100
		High	50,900	5.87	4.47	8700	11,400
Single household	30	Low	257,800	33.45	25.59	7700	10,100
		Middle	306,800	35.28	28.25	8700	10,900
		High	376,500	36.51	29.81	10,300	12,600
	65	Low	138,600	14.76	11.08	9400	12,500
		Middle	175,900	16.23	12.64	10,800	13,900
		High	228,200	17.34	13.69	13,200	16,700
	85	Low	47,800	4.84	3.52	9900	13,600
		Middle	62,300	5.44	4.09	11,500	15,200
		High	75,800	5.87	4.47	12,900	16,900

*Note:* Incremental costs and health effects are the result of a hypothetical intervention in which a death at a certain age is prevented at zero intervention costs. Incremental costs and health effects are the average of men and women at a particular age. Costs are expressed in EURO adjusted for 2017 prices. Costs were discounted at a 4% annual rate and health effects at a 1.5% annual rate.

Abbreviations: ICER, incremental cost effectiveness ratio; QALY, quality‐adjusted life year.

## 4 CONCLUSION AND DISCUSSION

Although the impact of including costs of non‐medical consumption on the *overall level* of the ICER is substantial, the differences in impact *across* educational groups are smaller. This is importantly related to two issues: (i) lower educated persons enjoy their added life‐years in a lower quality of life than higher educated, and (ii) lower educated persons more often form a single‐person household at an old age, which implies relatively high additional consumption costs compared to people in a multi‐person household. Especially this latter effect turned out to mitigate the socio‐economic differences induced by including the costs of non‐medical consumption. Finally, it needs to be noted that these influences also need to be viewed in relation to the inclusion of other costs (such as productivity costs) which may, on average, have opposite distributional effects.

Our findings are in line with previous studies that investigated the costs of non‐medical consumption (Kruse et al., 2012; Manns et al., 2003; Meltzer, 1997; Meltzer et al., 2000). The observation that consumption declines at older ages may be related to changing preferences and opportunities, but also to liquidity constraints: consumption seems to strongly follow the age pattern of disposable income (Alessie & Ree, 2009). This might also explain the differences in the age profiles of consumption between education groups (Fernández‐Villaverde, J., Krueger, D., 2007). Moreover, Gyrd‐Hansen suggested that the hump‐shaped pattern of consumption may also be explained by lower marginal utility from consumption for people in lower health (Gyrd‐Hansen, 2016).

An important and novel finding from this study is that the large differences in consumption between educational classes are mitigated due to concurrent differences in quality of life and household size. Differences in the probability to live in a multi‐person household were observed throughout the life‐course in different groups. Specifically, lower educated people live in a multi‐person household earlier than the other two education groups, but the peak in household size also occurs at an earlier age for the low educated. As we do not have data regarding the type of relationships within these households, several explanations for the observed differences are possible and these may also vary by age. Socioeconomic status for instance has been found to have an indirect effect on divorce rates through earlier marriages and worse economic status (Clarke & Berrington, 1998). Furthermore, differences in life expectancy between lower and higher educated might also explain why at old ages lower educated people are single more often (Gheorghe et al., 2016).

Although decisions about the availability of technologies are usually made at a fairly aggregate population level without distinguishing groups based on SES, it has been well documented that some health problems are relatively common in lower SES groups (Smith 1999: Cutler & Lleras‐Muney 2010). This makes distributional consequences of inclusion of particular costs increasingly relevant.

Summarizing, this paper provided empirical estimates of non‐medical survivor costs and an indication of the distributional consequences of including them in economic evaluations of life prolonging interventions. The current practice of including production gains but excluding future non‐medical costs not only has no economic rationale, but potentially also introduces socio‐economic inequalities in health following resource allocation decisions based on economic evaluations. Including future non‐medical costs may somewhat reduce the resulting socio‐economic inequalities. Estimates and methods described in this paper, facilitate their inclusion in economic evaluations and provide insight in the consequences of doing so. Given that influential guidelines like the US guidelines advocate their inclusion, this seems particularly useful and more research on theory, methods, estimates, and consequences of including these costs seems warranted.

## CONFLICT OF INTEREST

Mr. Kellerborg has nothing to disclose. Dr. Brouwer reports grants from the European Union in the H2020 programme, during the conduct of the study. Dr. Versteegh reports that he is director of the institute for Medical Technology Assessment (iMTA) which conducts contract research for public and private sector bodies. The institute is 100% owned by Erasmus University of Rotterdam and dr Versteegh does not receive any personal fees from collaborations with the private sector. Dr. Wouterse has nothing to disclose. Dr. van Baal has nothing to disclose.

## Supporting information

Supplementry Material S1

Supplementry Material S2

## Data Availability

The data that support the findings of this study are available from the corresponding author upon reasonable request.
