# Supplementary material for: Distributional consequences of including survivor costs in economic evaluations
Source: Health Econ. 2021 Jul 30;30(10):2606–13. doi: 10.1002/hec.4401 (PMC9292358; doi:10.1002/hec.4401)
Supplement: Supplementary file 1 — Supplementry Material S1 [file HEC-30-2606-s001.docx]

# Appendix

## More information on the data

Although educational attainment of other household members was also surveyed, the response for that question was much lower for multi person households and not relevant for single person households. Educational level in the Dutch budget survey is divided in four categories; lowest, low, middle and high based on the International Standard Classification of Education (ISCED). The lowest education level consists of individuals who only had a degree for the primary school. The low education level consists of individuals with a lower vocational education degree or those who completed high school (MAVO) or until year 3 of secondary science education (LBO, VMBO). The middle education level consists of individuals who completed general secondary education (HAVO), secondary science education (VWO) or secondary vocational education (MBO). The high category consists of individuals who completed higher professional education (HBO) or university (College degree). Due to few observations in the lowest category, we pooled the lowest and low category, resulting in three categories of educational attainment; low, middle and high. We included only those households whose head is older than 25 as on average individuals educate themselves until the age of 25 (77 households were excluded because of this). After these exclusions the sample consists of households who have answered to all the necessary questions with an age of 25 or higher which resulted in a sample size of 3,059 households.

Age was defined as the age of the head of the household. It was measured on an interval scale except for the end of the age spectrum. Ages of 80 and above were in the survey categorized as 80. We assigned the age of 82.5 to individuals in that category based on mean age of respondents above 80 in previous budget surveys (as in (Kellerborg et al., (submitted)). As the budget survey was not entirely representative for the Dutch population of households, we used sample weights provided by Statistics Netherlands. As the sample weights of Statistics Netherlands were partly determined by age and education, we centered these weights to 1 for each age and educational class strata.

## Additional results

##
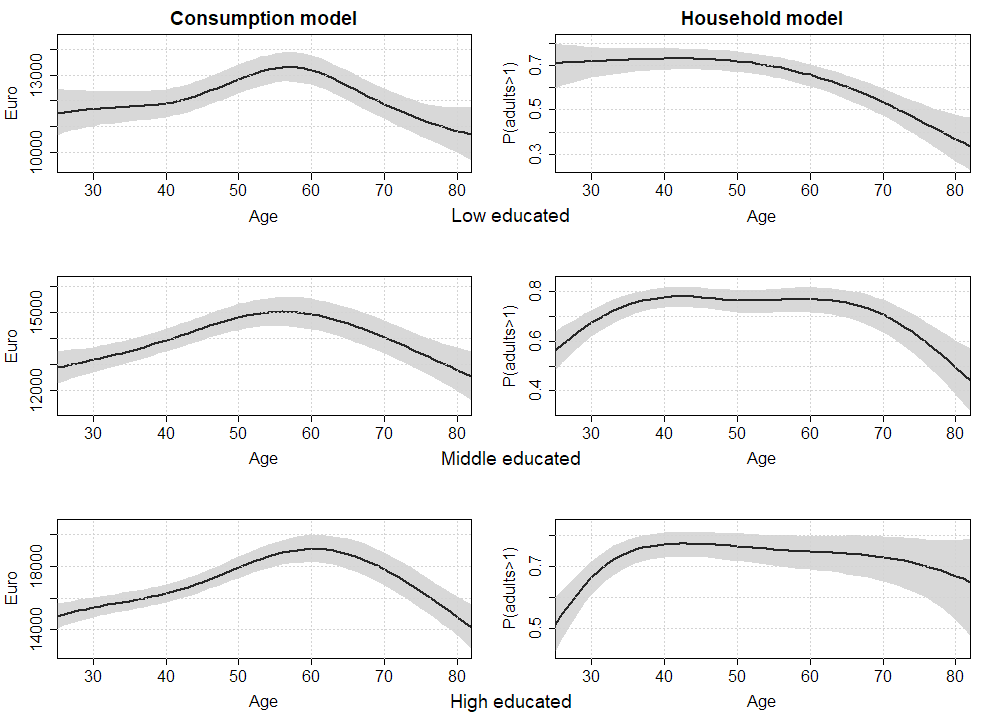


Figure S1. Consumption model predictions (left) by education and age, and household model predictions by education and age (right) including 95% prediction intervals.
